# Diet quality and eating behaviors in Brazilian medical undergraduates: associations with quality of life, sleep, and physical activity

**DOI:** 10.3389/fpubh.2026.1806572

**Published:** 2026-04-09

**Authors:** Pâmella Pollyanna B. C. Costela, Mariana Salles Kehl, Alexandre Sizilio, Renata Kobayasi, Rosimeire A. Q. Soares, Andred Meireles de Almeida Silva, Gustavo Nogueira Nóbrega, Mariana M. M. T. França, Daniel T. A. Espírito Santo, Marcelo Arruda Candido, Aviad Haramati, Patricia Tempski, Mílton A. Martins

**Affiliations:** 1 Center for the Development of Medical Education, Faculty of Medicine, University of São Paulo, São Paulo, Brazil; 2 Center for Innovation and Leadership in Education (CENTILE), Georgetown University School of Medicine, Washington, DC, United States

**Keywords:** medical students, diet quality, quality of life, sleep, physical activity

## Abstract

**Background:**

Medical students are exposed to behavioral risk factors, including unhealthy dietary practices, that may compromise health and wellbeing, particularly quality of life, sleep, and physical activity. We examined diet quality and eating practices, and their associations with these outcomes in Brazilian medical undergraduates.

**Methods:**

In this cross-sectional study, 105 students from a large public medical school completed validated assessments of diet quality, quality of life, sleep, and physical activity. Anthropometry and bioimpedance were obtained under standardized procedures. Group differences by diet quality categories were tested using parametric tests. Multivariable linear regression models adjusted for sex, age, and academic period were conducted. Effect sizes (η^2^, Cohen's d) and 95% CIs are reported. Significance: two-sided α = 0.05.

**Results:**

Participants had a mean age of 22.2 ± 3.4 years (52.4% male, 44.8% female, 2.9% other/undeclared). Diet quality classified 31.4% with < 31 points, 37.1% with 31–41 points, and 31.4% with >41 points. Healthier diet profiles were associated with higher quality-of-life scores in Psychological (*p* = 0.012, η^2^ = 0.083), Social relationships (*p* = 0.006, η^2^^2^ = 0.095), and Environment (*p* = 0.008, η^2^ = 0.091) domains. Sleep quality showed a marginal association (*p* = 0.050). Physical activity increased across diet strata (vigorous *p* = 0.003, η^2^ = 0.109; total *p* = 0.001, η^2^ = 0.121). Multivariable regression confirmed that associations with WHOQOL Psychological, Environment, IPAQ Vigorous, and IPAQ Total persisted after adjustment. No differences for DASS-21 or bioimpedance indices (all *p* ≥ 0.109).

**Conclusions:**

Healthier dietary practices as diet quality, meal planning, and food choices — were associated with better quality of life, physical wellbeing, and higher physical activity. These findings may inform campus-level health promotion and student support strategies.

## 1 Introduction

Human eating behavior is a multifaceted phenomenon encompassing biological, social, cultural, symbolic, and historical dimensions. Over recent decades, profound changes in dietary practices have been observed worldwide, primarily driven by the industrialization and globalization of food systems. The gradual replacement of fresh and minimally processed foods with ultra-processed foods (UPFs) has characterized the so-called “nutritional transition,” carrying deleterious implications for public health and population quality of life.

The NOVA classification (1, 2) provides a conceptual framework for understanding how industrial food processing impacts human health. Ultra-processed foods industrial formulations containing little to no whole food are engineered to be hyper-palatable and commercially profitable while being nutritionally unbalanced. Epidemiological evidence has linked higher UPF consumption to obesity, cardiovascular diseases, and mental health disorders (3–6). However, it is important to distinguish between studies that directly quantify UPF intake and those that assess overall diet quality through behavioral instruments capturing adherence to dietary guidelines without directly measuring specific food categories.

Brazil is no exception to this trend. Data from the VIGITEL Survey (7) and demonstrate a marked increase in UPF consumption, often at the expense of fresh foods and home-prepared meals, particularly among urban populations and vulnerable groups such as students. Within this context, medical students represent a population of particular interest. This group frequently reports inadequate dietary habits, low engagement in physical activity, sleep deprivation, and diminished subjective wellbeing. Paradoxically, these same individuals – future healthcare providers, frequently fail to integrate into their own lives the very principles of healthy living that they will be expected to advocate professionally.

The Dietary Guidelines for the Brazilian Population (8) recommend prioritizing fresh or minimally processed foods, a recommendation informed by the NOVA framework. Instruments developed to assess adherence to these guidelines, such as the “How is your diet?” questionnaire (4), evaluate diet quality through behaviors, meal planning, household organization, and food choices, rather than quantifying specific food group intake. Our primary objective was to investigate the relationship between diet quality and eating practices and multiple dimensions of health and wellbeing among medical students. We hypothesized that students with healthier eating practices would report better quality of life and sleep quality and would engage in higher levels of physical activity.

## 2 Materials and methods

### 2.1 Study design

This cross-sectional study was conducted at a large public medical school in São Paulo as part of the project “Health and Quality of Life of Brazilian Medical Students and Their Relationship with Professional Training” (São Paulo Research Foundation - FAPESP; Grant No. 2019/13850-9). Data were collected between 10 January 2022 and 30 June 2024. Ethical procedures are described in Section 2.6.

### 2.2 Participants

Eligible participants were medical students of both sexes, aged ≥18 years formally enrolled at the University of São Paulo. Exclusion criteria: students who did not complete all procedures. Sampling was non-probabilistic and convenience-based. No *a priori* sample size calculation was performed; the sample of 105 reflects total enrollment achieved during the collection period as convenience sample

### 2.3 Data collection

Participants attended in-person sessions at our research laboratory, scheduled by prior appointment. After reading and signing the informed consent form, participants completed validated psychometric instruments and underwent anthropometric assessments and objective health measurements. Data collection was conducted by trained researchers (senior medical students, physicians, and research assistants) who received standardized training on instrument administration and anthropometric measurement.

### 2.4 Instruments and variables

#### 2.4.1 Diet quality/dietary habits

Dietary patterns were assessed using the validated questionnaire (6) “How is your diet?”, composed of 24 items rated on a four-point Likert scale (ranging from “never” to “always”) Supplementary Table S1. The scale measures four dimensions: meal planning, household organization, eating behaviors, and food choices. The total score ranges from 0 to 72 and classifies participants into three categories: < 31 points (low), 31–41 points (intermediate), and >41 points (high diet quality). The validated “How is your diet?” questionnaire (6), 24 items on a four-point Likert scale. Cronbach's α = 0.793.

#### 2.4.2 Anthropometry and body composition

Body composition was measured via bioelectrical impedance analysis using the InBody 120^®^ device (multifrequency, segmental, tetrapolar), providing estimates of body weight, lean mass, fat mass, and body fat percentage. Height was measured with a digital stadiometer (HM-210D model) under standardized conditions (upright posture, barefoot, without head adornments).

#### 2.4.3 Physical Activity (IPAQ-SF)

Physical activity was evaluated using the International Physical Activity Questionnaire - Short Form (IPAQ-SF): a self-reported instrument that collects data on the frequency, duration, and intensity of daily physical activities. This questionnaire was translated and validated to Brazilian Portuguese (9).

#### 2.4.4 Sleep (PSQI)

Sleep was assessed using the Pittsburgh Sleep Quality Index (PSQI-BR), a 19-item instrument evaluating seven domains; total scores range from 0 to 21, with higher scores indicating poorer sleep quality. This questionnaire was translated and validated to Brazilian Portuguese (10).

#### 2.4.5 Quality of life (QoL)/(WHOQOL-BREF)

Quality of life was evaluated using the WHOQOL-BREF questionnaire, that consists of 26 items clustered in four domains: environment, psychological, social relationships and physical health. Answers are given on a 5-point Likert scale and points within each domain are linearly transformed to a score from 0 to 100, and higher scores represent better QoL. This questionnaire was translated and validated to Brazilian Portuguese (11).

#### 2.4.6 Mental health symptoms (DASS-21)

Mental health symptoms were evaluated using the Depression, Anxiety, and Stress Scale (DASS-21), a 21-item scale comprising three subscales for depression, anxiety, and stress. This questionnaire was translated and validated to Brazilian Portuguese (12). Cronbach's α = 0.798.

### 2.5 Statistical analysis

Statistical analyses were performed using SPSS (IBM SPSS Statistics), version 22. Two-sided significance was set at α = 0.05 (*p* < 0.05). Normality were assessed by Shapiro Wilk. Sex differences were assessed using independent-samples *t*-tests. Comparisons across diet quality categories < 31, 31–41, and >41 points) were conducted using one-way analysis of variance (ANOVA) followed by Bonferroni-adjusted *post hoc* tests and effect sizes were calculated with η^2^ (ANOVA), Cohen's d (pairwise). The sensitivity analyses were carried with Kruskal-Wallis for non-normal variables. We made Multivariable linear regression for significant outcomes, adjusting for sex, age, and academic period (reference: low diet quality) and 95% Cis were provided. Each participant contributed one measurement set with no pseudoreplication. Considering missing data we had only one participant missing bioimpedance, no imputation needed.

#### 2.5.1 Bias mitigation

To minimize information bias, all data collectors underwent standardized training, validated instruments were employed throughout, and anthropometric measurements were obtained using objective, standardized techniques. To reduce the risk of social desirability bias, all instruments were self-administered in a private setting, and responses were de-identified to ensure participant confidentiality

### 2.6 Ethics

This study was approved by the Research Ethics Committee of the University of São Paulo Medical School (CAAE 56043522.9.0000.0068). All participants provided written informed consent prior to enrolment and data collection. Participation was voluntary and could be discontinued at any time without penalty. Data were coded and analyzed in de-identified form to ensure confidentiality, and study materials were stored securely with access restricted to the research team.

## 3 Results

The sample comprised 105 medical students, with a mean age of 22.2 years (*SD* = 3.4; range: 18–36), of whom 52.4% identified themselves as males (*n* = 55), 44.8% as females (*n* = 47), and 2.9% (*n* = 3) identified with another gender or chose not to disclose. Regarding their stage of training, 50.5% of participants were in the first two years of the program (basic cycle), 39% in the third or fourth years (clinical cycle), and 10.5% in the final two years (medical internship/clinical clerkship).

In terms of dietary patterns, 83.8% self-identified as omnivores, while 10.5% reported following an ovolactovegetarian diet, 1% an ovo-vegetarian diet, and 3.8% adhered to a strict vegetarian diet. Regarding dietary quality, as assessed by the “How is your diet?” questionnaire, 31.4% (*n* = 33) scored < 31 points; 37.1% (*n* = 39) scored 31–41 points; and 31.4% (*n* = 33) scored >41 points, indicative of a healthy dietary pattern.

Table 1 shows the values of WHOQOL-BREF, DASS-21, PSQI and IPAQ questionnaires, comparing male and female medical students. IPAQ results were transformed into MET-min/week (metabolic equivalent of task minutes per week). We did not observe significant differences when male and female values were compared. DASS-21 scores indicated overall low-to-moderate symptom levels across depression, anxiety, and stress subscales. Considering bioimpedance values, as expected, males had more muscle mass and less body fat than females.

**Table 1:** Results of the questionnaires and bioimpedance values.

Instruments	Domains	Females *n* = 47	Males *n* = 55	*P*-value	Cohen d
		mean (SD)	mean (SD)		
WHOQOL-BREF	Physical	62.9 (15.2)	64.5 (16.0)	0.616	−0.10
	Psychological	59.3 (13.4)	63.5 (15.7)	0.155	−0.29
	Social relationships	69.5 (18.4)	66.5 (22.5)	0.470	0.14
	Environment	64.9 (14.2)	65.3 (17.0)	0.901	−0.03
Sleep quality	Epworth	9.8 (3.9)	9.5 (4.0)	0.654	0.08
	Pittsburg (PSQI)	6.4 (2.8)	6.7 (3.1)	0.557	−0.10
IPAQ (MET)	Walking	680.6 (715.3)	524.4 (463.7)	0.188	0.26
	Moderate exercise	562.6 (529.8)	452.7 (517.1)	0.294	0.21
	Vigorous exercise	2,567.7 (3,572.6)	1,904.7 (1,936.4)	0.258	0.24
	Total	3,810.9 (3,679.6)	2,881.9 (2,279.1)	0.137	0.31
Bioimpedance	Weight (Kg)	61.1 (11.8)	73.4 (14.5)	0.000^*^	−0.92
	Body fat mass	18.0 (7.9)	15.0 (9.0)	0.081	0.35
	Muscle mass	23.9 (4.2)	33.4 (6.6)	0.000^*^	−1.69
	Body mass index	22.8 (4.1)	24.0 (4.0)	0.150	−0.30
	Body fat	27.9 (8.1)	19.1(9.3)	0.000^*^	1.00
DASS	Depression	8.6 (7.8)	10.0 (10.8)	0.463	−0.15
	Anxiety	7.2 (7.2)	6.4 (6.9)	0.555	0.11
	Stress	16.0 (7.7)	13.8 (9.5)	0.206	0.25

MET, metabolic equivalent of task; SD, standard deviation; WHOQOL-BREF, World Health Organization Quality of Life – abbreviated version; PSQI, Pittsburgh Sleep Quality Index; IPAQ, International Physical Activity Questionnaire; DASS, depression, anxiety, and stress scale; BMI, body mass index.

t-Test ^*^P < 0.05.

QV, quality of life. Cohen's d: |d| < 0.2 = insignificant; 0.2–0.5 = little; 0.5–0.8 = medium; ≥ 0.8 = large.

Table 2 shows the comparisons of the results of the three groups of students considering the results of the questionnaire of nutritional habits. Students with better dietary practices showed significantly higher scores in WHOQOL Psychological, Social, and Environment. Students with healthier eating patterns also exhibited higher physical activity with higher overall and vigorous exercise IPAQ scores. We did not observe significant differences concerning mental health and bioimpedance scores.

**Table 2:** Comparisons of the three groups of medical students according to the results of the questionnaire of nutritional habits.

Instruments	Domains	Questionnaire of nutritional habits (dietary quality and habits) (mean ± SD)			*P*-value	η^2^ parcial
		Low <31 (*n* = 33)	Intermediate 31–41 (*n* = 39)	High >41 (*n* = 33)		
WHOQOL-BREF	Physical	61.9 (15.4)	62.1 (16.3)	69.0 (14.8)	0.103	0.044
	Psychological	58.8 (15.2)	59.0 (13.4)	67.9 (13.9)	0.012^*^	0.083
	Social relationships	67.2 (17.7)	61.8 (22.5)	77.0 (18.6)	0.006^*^	0.095
	Environment	62.2 (16.5)	61.7 (15.1)	72.0 (13.0)	0.008^*^	0.091
Pittsburgh (PSQI)	–	6.6 (3.4)	7.3 (2.9)	5.6 (2.1)	0.050	0.057
IPAQ (MET)	Walking	525.3 (574.5)	565.1 (534.0)	685.5 (665.7)	0.517	0.013
	Moderate exercise	429.1 (444.4)	515.0 (524.5)	567.3 (598.1)	0.561	0.011
	Vigorous exercise	1,230.3 (2,091.3)	1,925.1 (2,655.9)	3,481.2(3,123.6)	0.003^*^	0.109
	Total	2,184.6 (2,186.2)	3,005.2 (2,987.3)	4,734.0 (3,193.9)	0.001^*^	0.121
Bioimpedance	Body fat mass	18.3 (8.8)	14.0 (7.3)	16.5 (9.3)	0.109	0.043
	Skeletal muscle mass	28.8 (7.7)	28.7 (6.6)	29.8 (7.8)	0.794	0.004
	Body mass index	24.2 (4.4)	22.4 (3.4)	23.7 (4.3)	0.133	0.039
	Body fat	25.8 (9.5)	21.1 (9.8)	22.3 (9.5)	0.114	0.042
DASS	Depression	11.3 (10.9)	9.9 (9.8)	6.8 (6.7)	0.141	0.038
	Anxiety	7.0 (6.1)	7.3 (8.2)	5.6 (6.1)	0.576	0.011
	Stress	15.4 (8.8)	14.5 (8.8)	14.2 (8.7)	0.844	0.003

MET, metabolic equivalent of task; SD, standard deviation; WHOQOL-BREF, World Health Organization Quality of Life – abbreviated version; PSQI, Pittsburgh Sleep Quality Index; IPAQ, International Physical Activity Questionnaire; DASS, depression, anxiety, and stress scale; BMI, body mass index.

* statistically significant difference. ANOVA with Bonferroni correction.

### 3.1 *Post-hoc* comparisons

Bonferroni-corrected *post-hoc* comparisons revealed consistent advantages for the high diet quality group across multiple outcomes. For WHOQOL Psychological domain, the high group scored significantly higher than both the low (*p* = 0.041) and intermediate groups (*p* = 0.021). In the WHOQOL Social domain, a significant difference was observed between the high and intermediate groups (*p* = 0.008). Similarly, WHOQOL Environment scores were higher in the high group compared to both the low (*p* = 0.029) and intermediate groups (*p* = 0.009). Physical activity levels also differed significantly, with the high diet quality group presenting greater vigorous activity (*p* = 0.003) and total activity (*p* = 0.001) compared to the low group. Results provided in Supplementary Table S2.

### 3.2 Multivariable regression

In multivariable linear regression models adjusted for sex, age, and academic period (*n* = 102), high diet quality independently predicted better scores in WHOQOL Psychological (β = 9.56, *p* = 0.009), WHOQOL Environment (β = 10.77, *p* = 0.005), IPAQ Vigorous activity (β = 2,199, *p* = 0.001), and IPAQ Total activity (β = 2,453, *p* = 0.001). WHOQOL Social domain approached but did not reach statistical significance (β = 9.34, *p* = 0.067), and sleep quality as measured by the PSQI was not significantly associated with diet quality after adjustment (β = −0.85, *p* = 0.260). Full regression outputs are provided in Supplementary Table S3.

### 3.3 Sensitivity analyses

Non-parametric *Kruskal-Wallis*-tests confirmed all findings from the one-way ANOVA analyses, supporting the robustness of the results. Outcomes that were non-significant under parametric testing remained non-significant under the Kruskal-Wallis approach. Detailed results are presented in Supplementary Table S2.

## 4 Discussion

The findings indicate an association between healthier eating practices as assessed by a validated instrument based on the Dietary Guidelines for the Brazilian Population (8) and higher quality of life and physical activity among medical students.

It is important to note that our instrument assesses adherence to dietary guidelines through behavioral dimensions rather than directly quantifying UPF intake. The associations reflect relationships between overall diet quality and health outcomes, not direct effects of specific food categories (13, 14). In our sample, students with higher scores on the “How is your diet?” questionnaire also exhibited higher levels of physical activity, better sleep quality, and elevated scores in the physical and psychological domains of the WHOQOL-BREF, suggesting greater self-regulation and engagement in health-promoting self-care behaviors. Studies have shown that inadequate eating habits, sedentary lifestyles, and excess weight negatively impact the quality of life of medical and health science students, often associated with intense academic workload, time constraints, and high stress levels. (15–19).

Physical activity showed medium effects (IPAQ Total η^2^ = 0.121; Vigorous η^2^ = 0.109), and WHOQOL domains showed medium effects, all confirmed in regression To increase the proportion of adults who practice physical activity regularly, it is very important that medical students, residents and physicians in general provide adequate counseling to their patients. It has been shown in medical students that personal physical activity among medical students correlates with patient counseling frequency (20–24)Our finding that diet quality and physical activity are positively associated reinforces the importance of promoting integrated healthy lifestyle behaviors among future physicians. In addition, it has been shown that physicians and medical students with a normal body mass index (BMI) and who practice moderate and/or vigorous physical activity are more likely to feel confident about counseling their patients about physical activity than their colleagues who do not practice physical activity or are overweight (25). It is important that counseling about the practice of physical activity also includes its impact on QoL. However, there are few data concerning the relationship between QoL and physical activity in medical students and physicians.

Medical students are often immersed in clinical and care-related demands, affording them less flexibility for self-care and structured eating routines. This trend diverges from prior studies reporting progressive improvement in health behaviors throughout training, culminating in more conscious choices during the final years (26, 27). Such discrepancies underscore the importance of considering curricular and institutional specificities, as well as the cumulative effects of stress and workload in advanced stages of medical education.

The PSQI association was marginal (*p* = 0.050), did not survive regression (β = −0.85, *p* = 0.246), with small effect (η^2^^2^ = 0.057). This is suggestive but inconclusive. DASS-21 (η^2^^2^ ≤ 0.038) and bioimpedance (η^2^^2^ ≤ 0.043) showed no associations. Power was ≤ 0.47 for all. Effect sizes suggest clinically meaningful differences may not emerge even with larger samples. DASS-21 subscales showed relatively low mean scores across all groups, suggesting a possible floor effect. Regarding sleep, mean scores from both the PSQI; validated for the Brazilian population (10, 20) suggested insufficient or suboptimal patterns, although largely within non-pathological limits. Sleep deprivation among medical students is well-documented (28) and is associated, as observed here, with poorer quality of life and higher prevalence of stress and anxiety symptoms. These findings suggest that healthy eating behaviors may act as a protective factor, mitigating the deleterious impact of sleep deprivation and academic demands on students' overall health.

We previously evaluated a random sample of 1,350 medical students from 22 medical schools and compared the volume of leisure time physical activity and quality of life. Forty per cent of the medical students reported no leisure time physical activity (46.0 of females and 32.3% of males). In contrast, 27.2% were classified in the group of high volume of physical activity (21.0 of females and 34.2% of males). Using the group that reported no leisure time physical activity as the reference group, we observed a strong dose-effect relationship between the volume of leisure time physical activity and quality of life in both male and female medical students.

In this context, our findings reinforce the importance of future physicians developing not only healthy personal practices but also technical competencies in nutrition, as highlighted in the consensus by Eisenberg et al. (29), since self-care is a fundamental element both for promoting one's own health and for improving the quality of guidance provided to the population.

### 4.1 Strengths and limitations

This study has several strengths that enhance the interpretability and relevance of its findings to medical education. First, we adopted a comprehensive approach by integrating multiple dimensions of student wellbeing and health-related behaviors - diet quality, quality of life, sleep, physical activity, and body composition using validated instruments and standardized procedures. Second, the combination of self-reported measures with objective anthropometry and bioimpedance provides a more nuanced characterization of students' health profiles than studies relying exclusively on questionnaires. The analysis incorporated multivariable regression models adjusted for key covariates, non-parametric Kruskal-Wallis sensitivity analyses to confirm parametric findings, and effect size calculations throughout, enhanced the interpretability and reliability of the results.

Some limitations should also be acknowledged. The cross-sectional design precludes causal inference and does not allow temporal ordering between dietary practices and outcomes such as quality of life, sleep, and physical activity. The use of a non-probabilistic convenience sample from a single public medical school may limit generalizability to other institutions with different curricular structures, socioeconomic compositions, and campus environments.

No *a priori* power calculation was performed; *post-hoc* power estimates were adequate (≥0.80) for most significant outcomes, but fell below conventional thresholds for WHOQOL Psychological (0.78) and PSQI (0.60), suggesting these findings should be interpreted with caution. Although the study used validated questionnaires, key outcomes (e.g., physical activity, sleep quality, and mental health symptoms) were assessed through self-report and are therefore susceptible to recall bias and social desirability effects. In addition, because multiple outcomes and comparisons were examined, there is a potential risk of type I error; thus, *p*-values (particularly those near conventional thresholds) should be interpreted with caution and in conjunction with effect size patterns and plausibility. Finally, the diet instrument captures diet quality and related behaviors rather than detailed nutrient intake, which may limit the granularity of dietary characterization. Future studies should expand these findings using larger, multi-institutional samples and longitudinal designs to clarify directionality, strengthen causal interpretation, and inform targeted interventions within medical education.

Taken together, our findings support the view that diet-related behaviors -particularly lower reliance on ultra-processed foods - co-occur with more favorable profiles of wellbeing and health-related routines among medical students. By integrating validated measures of diet quality, quality of life, sleep, and physical activity alongside objective body composition indices, this study provides a clinically and educationally meaningful portrait of students' daily health practices. These results strengthen the rationale for institution-level strategies in medical education that move beyond individual responsibility, incorporating campus food environments, protected time for self-care, and curricular approaches that explicitly address the knowledge practice gap in nutrition and lifestyle counseling.

## 5 Conclusions

The present findings indicate that healthier dietary practices were associated with higher quality-of-life scores, better sleep quality, and greater physical activity among medical undergraduates. No significant differences were observed across diet quality strata for depression, anxiety, or stress symptoms, nor for bioimpedance indices. These results may inform campus-level health promotion strategies that integrate improvements in the food environment, protected time for self-care, and curricular approaches addressing the knowledge–practice gap in lifestyle counseling.

These results underscore the centrality of diet as a key determinant of overall health and a marker of self-care, particularly in populations vulnerable to psychological distress and self-neglect, such as medical students. Within the demanding context of medical education (marked by academic overload, performance pressure, and limited time) dietary patterns emerge as a privileged indicator of student wellbeing and their ability to regulate daily rhythms.

Accordingly, this study provides relevant empirical evidence to inform institutional strategies aimed at fostering healthier educational environments. Such strategies may include improving access to high-quality food on university campuses, creating designated spaces for rest and recuperation, and incorporating pedagogical initiatives that promote self-care, active listening, and wellbeing among future healthcare professionals.

Further research is warranted, particularly through larger samples and longitudinal designs, to assess the impact of targeted nutritional and behavioral interventions throughout medical training. The integration of institutional health policies with the ethical and technical training of students may contribute to the development of healthier physicians – professionals who are more attuned to their own limits and better equipped to promote comprehensive patient care.

## Data Availability

The raw data supporting the conclusions of this article will be made available by the authors, without undue reservation.
