# Supplementary material for: Diet quality and eating behaviors in Brazilian medical undergraduates: associations with quality of life, sleep, and physical activity
Source: Front Public Health. 2026 Apr 9;14:1806572. doi: 10.3389/fpubh.2026.1806572 (PMC13104623; doi:10.3389/fpubh.2026.1806572)
Supplement: Supplementary file 1 [file Table_1.docx]

Supplementary Material

# 1. Supplementary Tables

**Table S1. Questionnaire “How is your diet”?**

| When I eat small meals during the day, I usually have fruits or nuts | [A] [B] [C] [D] |
| --- | --- |
| When I choose fruits and vegetables, I prefer those that come from local farmers | [A] [B] [C] [D] |
| When I choose fruits and vegetables, I prefer those that are organic | [A] [B] [C] [D] |
| I usually carry some food with me in case I get hungry during the day | [A] [B] [C] [D] |
| I usually plan my daily meals | [A] [B] [C] [D] |
| I usually vary the consumption of beans among peas, lentils and chickpeas | [A] [B] [C] [D] |
| We commonly use whole-meal wheat flour at home | [A] [B] [C] [D] |
| I usually eat fruit at breakfast | [A] [B] [C] [D] |
| I usually eat breakfast/lunch/dinner at the table | [A] [B] [C] [D] |
| I try to eat slowly | [A] [B] [C] [D] |
| I usually buy foods at street market | [A] [B] [C] [D] |
| We share tasks which involve the preparation and consumption of foods at home | [A] [B] [C] [D] |
| I usually buy foods at street market | [A] [B] [C] [D] |
| I deal with personal issues during meal time, and therefore Iusually end up not eating anything | [A] [B] [C] [D] |
| I usually eat breakfast/lunch/dinner at my work’s or study’s desk | [A] [B] [C] [D] |
| I usually eat breakfast/lunch/dinner seated on the couchin the living room or in bed | [A] [B] [C] [D] |
| I usually skip at least one of the main meals (lunch or dinner) | [A] [B] [C] [D] |
| Iusuallyeatcandies,chocolatesand othersweets | [A] [B] [C] [D] |
| Iusually drink industrialized juices, such as those which are powdered or the ones that are packed in boxes, bottles or tins | [A] [B] [C] [D] |
| I usually go to fast-food restaurants or snack bars | [A] [B] [C] [D] |
| I often snack between meals | [A] [B] [C] [D] |
| I usually drink soft drinks | [A] [B] [C] [D] |
| Iusually take sandwiches, savoury snacks or pizza for lunch or dinner instead of freshly prepared dishes | [A] [B] [C] [D] |
| When I drink coffee or tea, I usually add sugar | [A] [B] [C] [D] |

A – Never; B – Rarely; C – Often; D – Always

**Table S2. Post-hoc power analysis and pairwise effect sizes (Cohen's d) by diet quality group**

| Instruments/Domains | | F | η² | Cohen f | Power (1−β) | Cohen's d Low–Inter | Cohen's d Low–High | Cohen's d Inter–High |
| --- | --- | --- | --- | --- | --- | --- | --- | --- |
| WHOQOL-BREF | Physical | 2.327 | 0.044 | 0.214 | 0.474 | 0.01 | 0.47 | 0.45 |
|  | Psychological | 4.591 | 0.083 | 0.300 | 0.779 | 0.01 | 0.62 | 0.66 |
|  | Social Relationships | 5.335 | 0.095 | 0.323 | 0.841 | -0.27 | 0.54 | 0.73 |
|  | Environment | 5.110 | 0.091 | 0.317 | 0.824 | -0.03 | 0.66 | 0.72 |
| PSQI | | 3.078 | 0.057 | 0.246 | 0.596 | 0.21 | -0.35 | -0.65 |
| DASS | Depression | 1.996 | 0.038 | 0.198 | 0.415 | -0.13 | -0.49 | -0.36 |
|  | Anxiety | 0.554 | 0.011 | 0.104 | 0.142 | 0.03 | -0.23 | -0.22 |
|  | Stress | 0.170 | 0.003 | 0.058 | 0.077 | -0.10 | -0.14 | -0.04 |
| IPAQ | Walking | 0.664 | 0.013 | 0.114 | 0.162 | 0.07 | 0.26 | 0.20 |
|  | Moderate MET | 0.582 | 0.011 | 0.107 | 0.147 | 0.17 | 0.26 | 0.09 |
|  | Vigorous MET | 6.242 | 0.109 | 0.350 | 0.896 | 0.29 | 0.85 | 0.54 |
|  | Total MET | 6.997 | 0.121 | 0.370 | 0.928 | 0.31 | 0.93 | 0.56 |
| Bioimpedance | Body mass index | 2.055 | 0.039 | 0.201 | 0.425 | -0.48 | -0.12 | 0.35 |
|  | Body Fat % | 2.217 | 0.042 | 0.208 | 0.454 | -0.48 | -0.36 | 0.13 |
|  | Body Fat Mass (kg) | 2.265 | 0.043 | 0.211 | 0.463 | -0.52 | -0.19 | 0.30 |
|  | Skeletal Muscle Mass (kg) | 0.231 | 0.005 | 0.067 | 0.086 | -0.01 | 0.13 | 0.15 |

η² = eta-squared. Cohen's d: positive values indicate higher mean in the higher diet quality group. Low = <31 points; Intermediate = 31–41; High = >41 from questionnaire of nutritional habits.

**Table S3. Multivariable linear regression models for significant outcomes (n = 102)**

| Instruments/Domains | Predictor | β | SE | t | p | 95% CI Lower | 95% CI Upper |
| --- | --- | --- | --- | --- | --- | --- | --- |
| WHOQOL Psychological | Intercept | 58.50 | 10.29 | 5.68 | < 0.001 | 38.07 | 78.92 |
|  | Diet: Intermediate | 0.27 | 3.45 | 0.08 | 0.939 | -6.59 | 7.12 |
|  | Diet: High | 9.56 | 3.58 | 2.67 | 0.009 | 2.45 | 16.67 |
|  | Sex (male) | 5.30 | 2.91 | 1.82 | 0.072 | -0.49 | 11.08 |
|  | Age | -0.14 | 0.46 | -0.30 | 0.766 | -1.04 | 0.77 |
|  | Academic period | 0.25 | 2.42 | 0.10 | 0.918 | -4.56 | 5.06 |
| WHOQOL Social Relationships | Intercept | 71.80 | 14.48 | 4.96 | < 0.001 | 43.07 | 100.54 |
|  | Diet: Intermediate | -6.57 | 4.86 | -1.35 | 0.179 | -16.21 | 3.07 |
|  | Diet: High | 9.34 | 5.04 | 1.85 | 0.067 | -0.66 | 19.35 |
|  | Sex (male) | -1.31 | 4.10 | -0.32 | 0.750 | -9.45 | 6.83 |
|  | Age | -0.13 | 0.64 | -0.20 | 0.843 | -1.40 | 1.15 |
|  | Academic period | -1.57 | 3.41 | -0.46 | 0.647 | -8.33 | 5.20 |
| WHOQOL Environment | Intercept | 55.01 | 10.86 | 5.07 | < 0.001 | 33.46 | 76.57 |
|  | Diet: Intermediate | -1.60 | 3.64 | -0.44 | 0.661 | -8.83 | 5.63 |
|  | Diet: High | 10.77 | 3.78 | 2.85 | 0.005 | 3.26 | 18.28 |
|  | Sex (male) | 2.74 | 3.08 | 0.89 | 0.375 | -3.36 | 8.85 |
|  | Age | 0.38 | 0.48 | 0.79 | 0.434 | -0.58 | 1.34 |
|  | Academic period | -4.53 | 2.56 | -1.77 | 0.080 | -9.60 | 0.55 |
| IPAQ Vigorous MET | Intercept | 4128.06 | 1930.50 | 2.14 | 0.035 | 296.05 | 7960.08 |
|  | Diet: Intermediate | 733.47 | 647.65 | 1.13 | 0.260 | -552.10 | 2019.04 |
|  | Diet: High | 2199.63 | 672.22 | 3.27 | 0.001 | 865.28 | 3533.98 |
|  | Sex (male) | -471.02 | 546.85 | -0.86 | 0.391 | -1556.51 | 614.48 |
|  | Age | -119.48 | 85.72 | -1.39 | 0.167 | -289.63 | 50.67 |
|  | Academic period | 65.50 | 454.47 | 0.14 | 0.886 | -836.62 | 967.61 |
| IPAQ Total MET | Intercept | 5974.53 | 2043.41 | 2.92 | 0.004 | 1918.40 | 10030.66 |
|  | Diet: Intermediate | 883.75 | 685.53 | 1.29 | 0.200 | -477.01 | 2244.50 |
|  | Diet: High | 2453.05 | 711.54 | 3.45 | 0.001 | 1040.66 | 3865.44 |
|  | Sex (male) | -741.36 | 578.84 | -1.28 | 0.203 | -1890.34 | 407.62 |
|  | Age | -155.02 | 90.73 | -1.71 | 0.091 | -335.12 | 25.08 |
|  | Academic period | 161.69 | 481.05 | 0.34 | 0.738 | -793.18 | 1116.57 |
| PSQI | Intercept | 3.22 | 2.10 | 1.54 | 0.128 | -0.94 | 7.38 |
|  | Diet: Intermediate | 0.68 | 0.70 | 0.96 | 0.339 | -0.72 | 2.07 |
|  | Diet: High | -0.85 | 0.73 | -1.17 | 0.246 | -2.30 | 0.60 |
|  | Sex (male) | 0.30 | 0.59 | 0.51 | 0.613 | -0.88 | 1.48 |
|  | Age | 0.15 | 0.09 | 1.59 | 0.115 | -0.04 | 0.33 |
|  | Academic period | -0.18 | 0.49 | -0.37 | 0.715 | -1.16 | 0.80 |

Reference category: Low diet quality (<31 points). Models adjusted for sex, age, and academic period (0 = basic cycle, 1 = clinical cycle, 2 = internship). n = 102 (participants with complete data on all covariates). β = unstandardized regression coefficient. SE = standard error. 95% CI = 95% confidence interval. MET = metabolic equivalent of task. PSQI = Pittsburgh Sleep Quality Index (included for completeness).

**Table S4. Distribution assumptions: Shapiro-Wilk normality test by group, Levene's test of homogeneity of variances, and Kruskal-Wallis nonparametric test**

| Instruments/Domains | | Shapiro-Wilk: Low | | Shapiro-Wilk: Intermediate | | Shapiro-Wilk: High | | Levene's p | Kruskal-Wallis H | Kruskal-Wallis p |
| --- | --- | --- | --- | --- | --- | --- | --- | --- | --- | --- |
|  |  | W | p | W | p | W | p |  |  |  |
| WHOQOL-BREF | Physical | 0.947 | 0.107 | 0.963 | 0.218 | 0.973 | 0.568 | 0.629 | 4.39 | 0.111 |
|  | Psychological | 0.969 | 0.447 | 0.970 | 0.368 | 0.983 | 0.880 | 0.955 | 8.17 | 0.017 |
|  | Social Relationships | 0.929 | 0.032* | 0.965 | 0.269 | 0.877 | 0.001* | 0.278 | 10.52 | 0.005 |
|  | Environment | 0.953 | 0.159 | 0.957 | 0.142 | 0.952 | 0.153 | 0.687 | 8.16 | 0.017 |
| PSQI | | 0.971 | 0.507 | 0.931 | 0.019* | 0.924 | 0.024* | 0.031* | 5.23 | 0.073 |
| DASS | Depression | 0.860 | 0.001* | 0.847 | < 0.001* | 0.864 | 0.001* | 0.262 | 3.19 | 0.203 |
|  | Anxiety | 0.897 | 0.004* | 0.824 | < 0.001* | 0.813 | < 0.001* | 0.388 | 1.26 | 0.532 |
|  | Stress | 0.966 | 0.389 | 0.939 | 0.034* | 0.962 | 0.296 | 0.895 | 0.53 | 0.768 |
| IPAQ | Walking | 0.664 | < 0.001* | 0.833 | < 0.001* | 0.790 | < 0.001* | 0.565 | 1.37 | 0.504 |
|  | Moderate MET | 0.854 | < 0.001* | 0.866 | < 0.001* | 0.839 | < 0.001* | 0.561 | 0.63 | 0.730 |
|  | Vigorous MET | 0.644 | < 0.001* | 0.646 | < 0.001* | 0.857 | 0.001* | 0.115 | 18.14 | < 0.001 |
|  | Total MET | 0.798 | < 0.001* | 0.713 | < 0.001* | 0.913 | 0.012* | 0.346 | 17.91 | < 0.001 |
| Bioimpedance | Body mass index | 0.955 | 0.190 | 0.951 | 0.088 | 0.881 | 0.002* | 0.576 | 4.07 | 0.131 |
|  | Body Fat % | 0.954 | 0.174 | 0.932 | 0.021* | 0.960 | 0.260 | 0.607 | 4.48 | 0.106 |
|  | Body Fat Mass (kg) | 0.935 | 0.048* | 0.943 | 0.048* | 0.859 | 0.001* | 0.800 | 4.61 | 0.100 |
|  | Skeletal Muscle Mass (kg) | 0.976 | 0.666 | 0.938 | 0.034* | 0.924 | 0.024* | 0.540 | 0.32 | 0.851 |
| Shapiro-Wilk W and p-values reported per diet quality group (Low = <31; Intermediate = 31–41; High = >41). * p < 0.05 (deviation from normality or heteroscedasticity). Levene's test: null hypothesis of equal variances across groups. Kruskal-Wallis H statistic and p-values confirm ANOVA findings for all outcomes with non-normal distributions. MET = metabolic equivalent of task | | | | | | | | | | |
